# Type-5 abomasal ulcer and omental bursitis in 14 cows

**DOI:** 10.1186/s13028-020-0501-1

**Published:** 2020-01-13

**Authors:** Ueli Braun, Christina Reif, Monika Hilbe, Christian Gerspach

**Affiliations:** 1 Department of Farm Animals, Vetsuisse Faculty, University of Zurich, Zurich, Switzerland; 2 Institute of Veterinary Pathology, Vetsuisse Faculty, University of Zurich, Zurich, Switzerland

**Keywords:** Abomasum, Cattle, Omental bursitis, Type-5 ulcer

## Abstract

**Background:**

Type-5 abomasal ulcer (U5) is a perforated ulcer that causes peritonitis limited to the omental bursa. This retrospective study describes the clinical and laboratory findings in 14 cattle with omental bursitis due to U5. The medical records of 14 cows aged 2.5 to 14.6 years (5.4 ± 3.1 years) with U5 were scrutinised.

**Results:**

The most common clinical findings were partial or complete anorexia (100%), abdominal guarding (100%), obtunded demeanour (93%), congested scleral vessels (79%), tachypnoea (71%), rumen atony (64%), diminished faecal output (64%), reduced skin surface temperature (64%) and fever (46%). Four (29%) cows had between one and four concomitant diseases. The most common abnormal laboratory findings were hypokalemia (71%), haemoconcentration (57%), metabolic acidosis (57%) and azotaemia (43%). All cows were euthanased; five immediately after the initial examination, one after exploratory laparotomy and eight after unsuccessful treatment. A diagnosis of U5 was made in all cows during postmortem examination.

**Conclusions:**

There is a need for improvement of the antemortem diagnosis of U5 because reliable differentiation of this disease from other conditions with a similar clinical presentation is currently not feasible.

## Background

Abomasal ulcers are grouped into four [1–5] or five types [6], depending on the author. Type-1 ulcer (U1) is a non-perforated superficial mucosal defect associated with minimal haemorrhage, and is further classified into four subtypes 1a to 1d [7]. Massive intraluminal haemorrhage caused by erosion of a major blood vessel is seen with type-2 ulcer (U2). Type-3 ulcer (U3) is a perforated abomasal lesion associated with localised peritonitis, and type 4 ulcer (U4) is a perforated lesion characterised by diffuse peritonitis because of contamination of the abdominal cavity with ingesta. More than one ulcer type can occur at the same time [8].

**Fig. 1:**
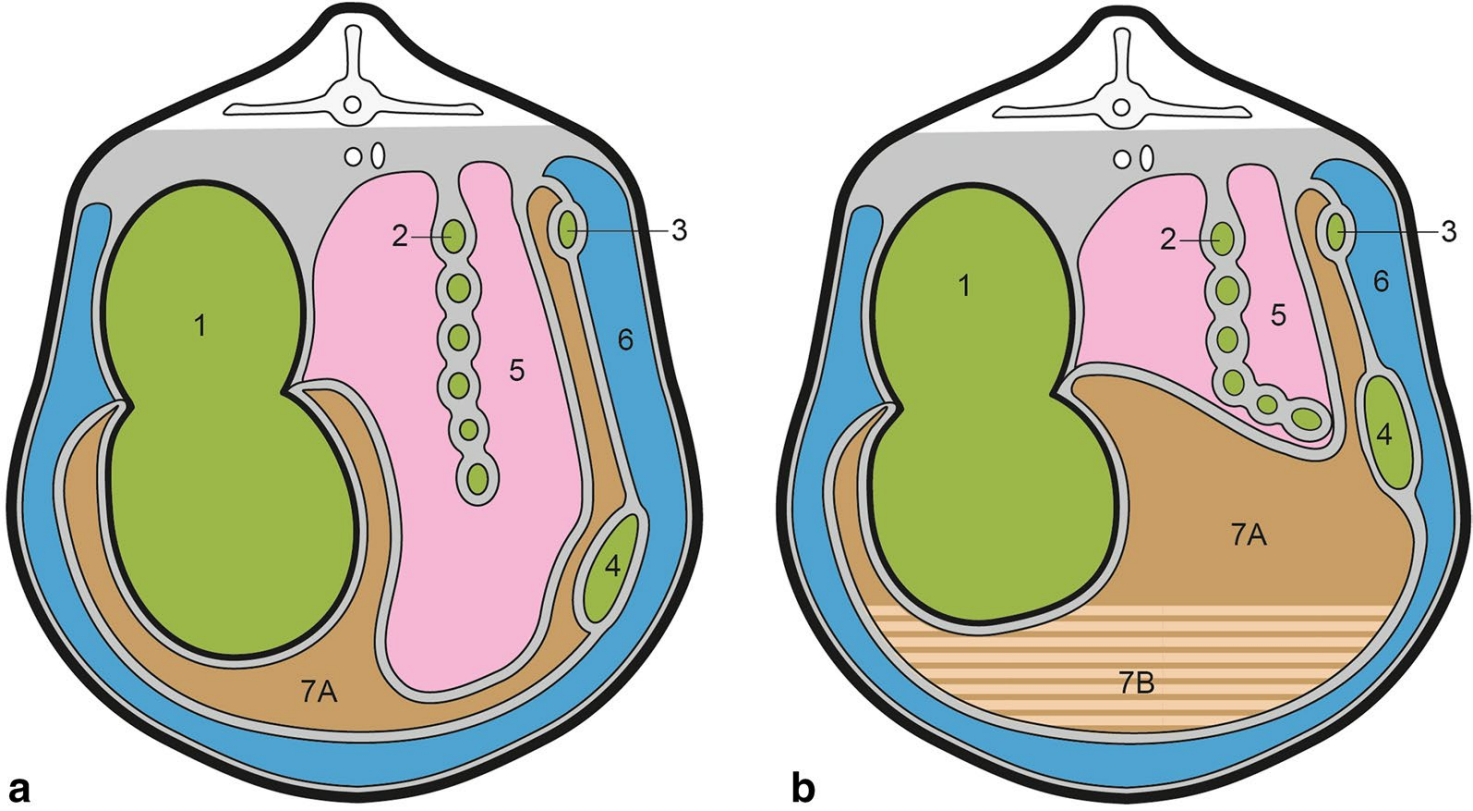
Cross section of the bovine abdomen. Illustration of a cross section of the bovine abdomen, modified after Hemmingsen [13]. **a** Normal findings, **b** Omental bursitis with empyema in the omental bursa. 1: Rumen; 2: Spiral colon; 3: Duodenum; 4: Abomasum; 5: Intestinal recess; 6: Peritoneal cavity; 7 A: Omental bursa; 7 B: Empyema in a cow with omental bursitis (hatched)

Abomasal perforation into the omental bursa causing omental bursitis was formerly classified as a sub-type of U3 [9] but is now known as type-5 ulcer (U5) [6]. The clinical and laboratory findings of cows with abomasal ulcer vary widely and were recently described in detail in 87 cows with U4 [10], in 145 cows with U2 [11] and in 60 cows with U3 [12]. Type-5 ulcer occurs when the perforation is in the left abomasal wall allowing the abomasal contents to leak into the omental bursa causing omental bursitis [13, 14] as shown in Fig. 1 [15, 16]. In contrast, perforation of the right abomasal wall leads to U4 with leakage of abomasal contents into the peritoneal cavity and diffuse peritonitis. Omental bursitis is characterised by peritonitis with a suppurative exudate, often with a fetid odour, and frequently accompanied by empyema in the omental sac or between the two serosal layers of the bursa [17]. There are few reports of omental bursitis in cattle [14–16, 18]. Other causes of omental bursitis include necrotising rumenitis, foreign body-induced reticular perforation [15] and spread of infection from umbilical disease, localised peritonitis or parametritis into the omental bursa [19]. The goal of this report was to add to the clinical understanding of U5 in cattle and to describe the clinical and laboratory findings in 14 cows with this condition.

## Methods

### Animals

This was a retrospective study of 14 cows that had been diagnosed with U5. The cows had been admitted to the Veterinary Teaching Hospital, University of Zurich, from January 1, 1991 to December 31, 2014. A definitive diagnosis of U5 was based on the results of postmortem examination. The results were described in detail in a dissertation [20]. The cows ranged in age from 2.5 to 14.6 years (mean ± sd = 5.4 ± 3.1 years). Breeds included Swiss Braunvieh (n = 7), Holstein–Friesian (n = 5) and Swiss Fleckvieh (n = 2). The duration of illness was < 2 days in 7 cows, 2 to 6 days in 4 cows and 7 to 14 days in 3 cows. Four cows had calved within 4 weeks of becoming ill.

### Clinical examination and laboratory analyses

The cows underwent a thorough clinical examination as described previously [10]. Blood samples were collected from all cows for determination of haematocrit, total leukocyte count and the concentrations of total protein, fibrinogen, serum urea nitrogen and potassium, a glutaraldehyde clotting test and venous blood gas analysis [10]. Urine was examined using a test strip (Combur^9^, Roche) and specific gravity was measured with a refractometer. A sample of rumen fluid was collected from ten cows for determination of the chloride concentration and assessment of colour, odour, consistency and pH. The methylene blue reduction time was also determined (data not shown).

### Ultrasonographic examination and abdominocentesis

The reticulum was examined ultrasonographically in ten cows, the abomasum in five and the abdomen in 12 as described [21]. Ultrasound-guided abdominocentesis and fluid analysis were carried out in seven cows in which abdominal fluid was seen [22]. The aspirated fluid was considered an exudate when at least one of the following criteria was met: specific gravity > 1.015, protein concentration > 30 g/l, cloudy appearance, malodourous and green discoloration.

### Treatment, euthanasia, postmortem examination and diagnosis

All cows were euthanased immediately after initial examination, exploratory laparotomy or unsuccessful treatment. Treatment included intravenous administration of a solution containing 50 g glucose and 9 g NaCl/l via an indwelling jugular vein catheter, antibiotics (penicillin G procaine, 12,000 IU/kg body weight (BW), Procacillin®, MSD Animal Health, or amoxicillin, 7 mg/kg BW, Clamoxyl®, Zoetis Switzerland) administered intramuscularly, and flunixin meglumine (1 mg/kg BW, Flunixin®, Biokema), ketoprofen (3 mg/kg BW, Rifen®, Streuli Pharma) or metamizole (35 mg/kg BW, Vetalgin®, MSD Animal Health) administered intravenously. Pentobarbital (Esconarkon, Streuli Pharma, 80 mg/kg BW) administered intravenously was used for euthanasia. All cows underwent postmortem examination, and a diagnosis of U5 was made when a perforated abomasal ulcer accompanied by omental bursitis was seen.

### Statistical analysis

The program IBM SPSS Statistics 22.0 was used for analysis. Frequencies were determined for each variable. The Wilk-Shapiro test was used to test the data for normality. Means ± standard deviations were calculated for normal data (respiratory rate, haematocrit, potassium) and medians for non-normal data (heart rate, rectal temperature, total leukocyte count, total protein, fibrinogen, urea, glutaraldehyde test time, pH, pCO_2_, HCO_3_^−^ and base excess of venous blood, urine pH, urine specific gravity). A value of P < 0.05 was considered significant.

## Results

### Clinical findings

**Fig. 2:**
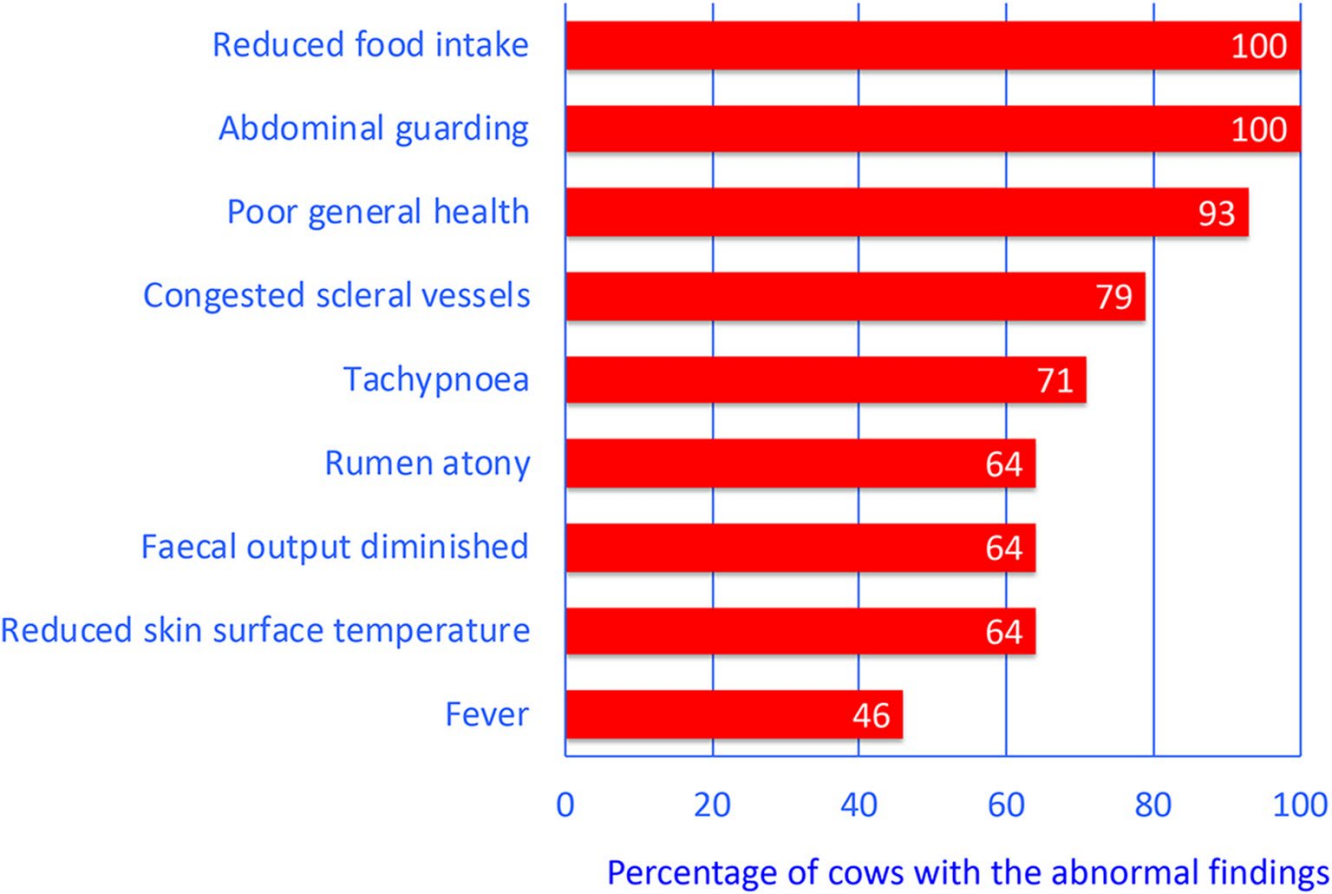
Abnormal clinical findings in 14 cows. Most common abnormal clinical findings in 14 cows with type-5 abomasal ulcer

The most common clinical findings were, in decreasing order of frequency, partial or complete anorexia (100%), abdominal guarding (100%), obtunded demeanour (n = 13 [93%]), congested scleral vessels (n = 11 [79%]), tachypnoea (n = 10 [71%]), rumen atony (n = 9 [64%]), decreased faecal output (n = 9 [64%]), reduced skin surface temperature (n = 9 [64%]) and fever (n = 6 [46%]) (Fig. 2, Table 1). One cow was recumbent on admission. In addition to abdominal guarding, signs of pain included arched back (n = 5 [36%]), bruxism (n = 3, 21%) and spontaneous grunting, muscle tremors and weight shifting (each n = 2, 14%). In eight cows (62%), all three tests for cranial abdominal pain (pinching of the withers, pole test and percussion of the abdominal wall over the region of the reticulum) were negative, and in five cows (38%), at least one test was positive. Ballottement and simultaneous auscultation (BSA) and/or percussion and simultaneous auscultation (PSA) were negative on the left side in 11 cows (79%) and on the right side in five (36%); in all other cows, one or both tests were positive. Faecal consistency varied from liquid to normal to drier than normal. Seven cows (50%) had diarrhoea and faecal colour was dark brown or black in three cows (21%). Transrectal examination showed reduced intra-abdominal pressure and crepitus in one cow (7%) each, ruminal distension in three (21%) and unclear findings in two others (14%).

**Table 1:** Clinical findings in 14 cows with type 5 abomasal ulcers

Variable	Finding	Number of cattle	%
Heart rate (n = 14, median = 74 bpm	Normal (60–80)	8	57
	Decreased (56–59) Increased (81–148)	1 5	7 36
Respiratory rate (n = 14, mean ± sd = 30 ± 8 breaths per min.)	Normal (16–25)	4	29
	Increased (26–45)	10	71
Rectal temperature (n = 13, median = 38.9 °C)	Normal (38.4–38.9)	5	39
	Decreased (37.5–38.3)	2	15
	Increased (39.0–39.4)	6	46
Rumen motility (n = 14)	Normal Decreased Absent	1 4 9	7 29 64
Foreign body tests(n = 13)	All negative Back grip positive^a^ Pole test positive^a^ Percussion of the reticulum positive^a^ At least one test positive	8 3 3 3 5	62 23 23 23 38
BSA and PSA on the left side (n = 14)	Both negative (normal) Only BSA positive	11 3	79 21
BSA and PSA on the right side (n = 14)	Both negative (normal) Only swinging auscultation positive Both positive	5 5 4	36 36 29
Faeces (n = 14)	Amount of feces decreased Faeces watery to loose Faeces dark to black	9 7 3	64 50 21
Rectal findings (n = 14)	Loss of negative pressure Crepitus	1 1	7 7

*Bpm* beats per minute, *BSA* Ballottement and simultaneous auscultation, *PSA* Percussion and simultaneous auscultation

^a^Positive: at least 3 of 4 tests elicited a grunt

### Laboratory findings (blood, urine, rumen fluid)

**Fig. 3:**
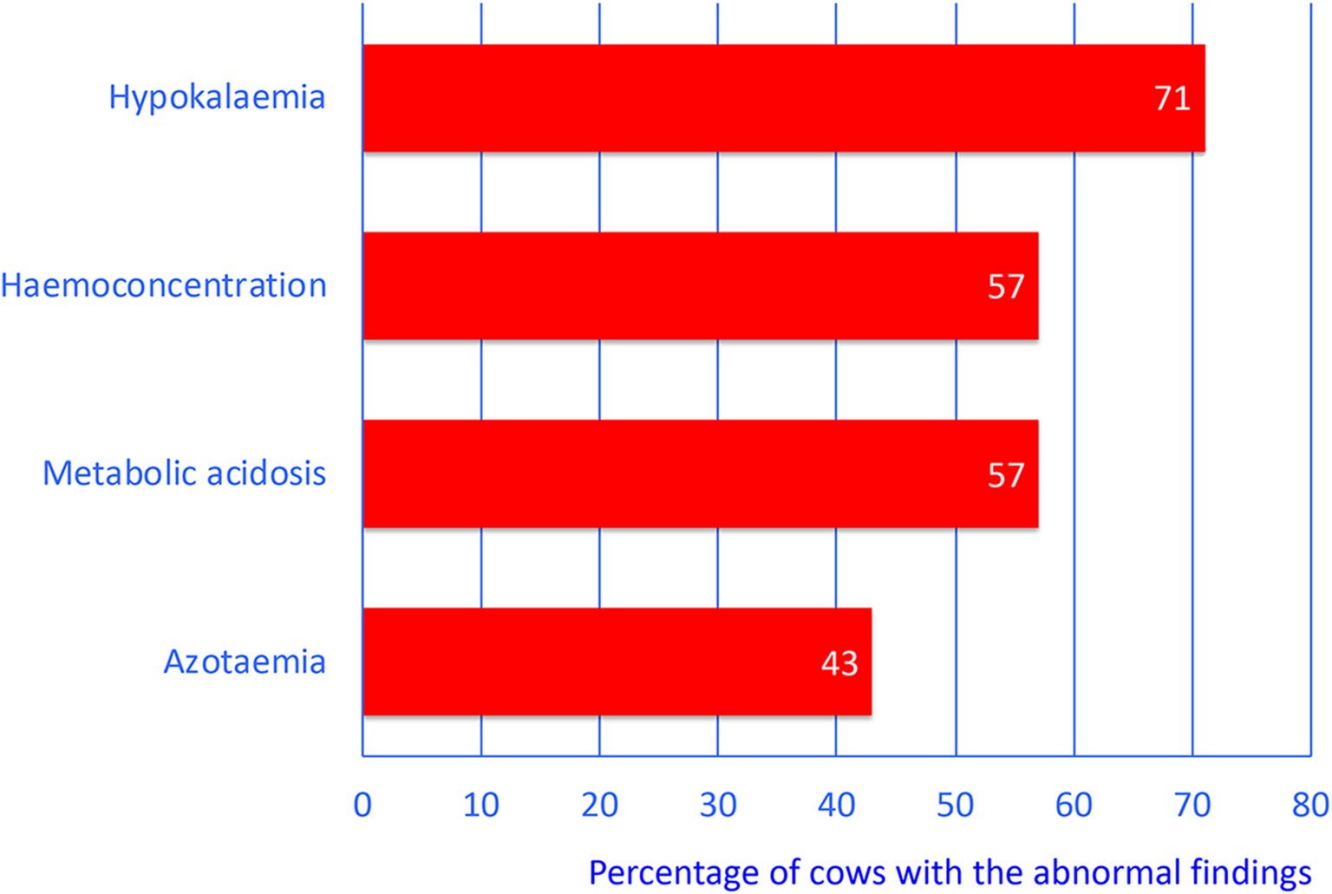
Abnormal blood variables in 14 cows. Most common abnormal blood variables in 14 cows with type-5 abomasal ulcer

The most common haematological and biochemical abnormalities were, in decreasing order of frequency, hypokalemia (n = 10 [71%]), haemoconcentration (n = 8 [57%]), metabolic acidosis (n = 8 [57%]) and azotaemia (n = 6 [43%]) (Fig. 3). Two cows (14%) had abnormal total leukocyte counts with leukopenia in one and leukocytosis in the other (Table 2). The total protein concentration was decreased in three cows (21%) and the fibrinogen concentration in two (14%), and in three cows (21%), the fibrinogen concentration was increased. The glutaraldehyde test time was shortened (< 10 min) in four cows (29%).

**Table 2:** Haematological and blood biochemical findings in 14 cows with type 5 abomasal ulcers

Variable (mean ± sd or median)	Finding	Number of cattle	Percent
Haematocrit (%) (n = 14, mean ± sd = 34.9 ± 8.7%)	Normal (30–35) Decreased (19–29) Increased (36–51)	2 4 8	14 29 57
White blood cell count (/µl) (n = 14, median = 6,100/µl)	Normal (5000–10,000) Decreased (4400–4999) Increased (10,001–25,400)	10 2 2	71 14 14
Total protein concentration (n = 14, median = 60 g/l)	Normal (60–80) Decreased (48–59)	11 3	79 21
Fibrinogen concentration (n = 14, median = 5.5 g/l)	Normal (4–7) Decreased (1–3) Increased (8–14)	9 2 3	64 14 21
Urea concentration (n = 14, median = 5.7 mmol/l)	Normal (2.4–6.5) Increased (6.6–23.8)	8 6	57 43
Potassium (n = 14, mean ± sd = 3.7 ± 0.9 mmol/l)	Normal (4.0–5.0) Decreased (2.0–3.9) Increased (5.1–5.9)	3 10 1	21 71 7
Glutaraldehyde test (n = 14, median = 10.0 min)	6.1 to 9.9 10 > 10	4 3 7	29 21 50

Six cows (43%) had haematuria with macroscopically normal urine (5 to 250 erythrocytes/µl), six cows (43%) had glucosuria (0.5 to 10 g/l), three cows (21%) had aciduria (pH < 6.5) and two cows (14%) had ketonuria (acetoacetate > 0.5 g/l and/or acetone > 0.4 g/l). Urine specific gravity was decreased (1.000 to 1.019) in three (23%) and increased (1.042 to 1.045) in two of 13 tested samples (15%). The chloride concentration of rumen fluid was increased (26 to 50 mmol/l) in six of ten tested samples (60%).

All seven samples of abdominal fluid were exudates. Four samples were yellow, two were green and one was brown. All samples were opaque and five were malodourous. Specific gravity ranged from 1.012 to 1.036 (1.027 ± 0.011) and the protein concentration from 10 to 62 g/l (median 44 g/l).

### Ultrasonographic findings

**Fig. 4:**
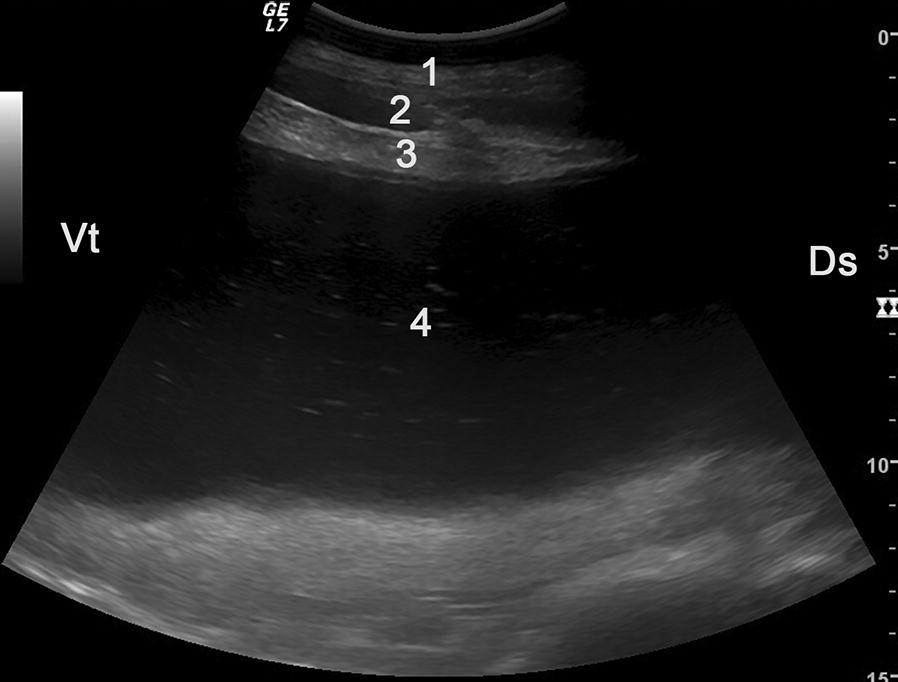
Ultrasonogram of the abdomen of a cow with omental bursitis caused by type-5 abomasal ulcer, imaged from the distal region of the 10th intercostal space on the left side. There is a small fluid accumulation in the peritoneal cavity and a large fluid accumulation in the omental bursa. The fluid in the omental bursa is characterized by echoic stippling indicative of microbial gas production. 1 Lateral abdominal wall of the left side, 2 Fluid in the peritoneal cavity, 3 Greater omentum, 4 Fluid in the omental bursa with echoic stippling, *Ds* Dorsal, *Vt* Ventral

The reticulum was elevated from the ventral abdominal wall in two of ten cases (20%), had an abnormal contour in four (40%) and decreased amplitude of contractions in three cows (30%) (Table 3). Reticular atony was diagnosed in four cows (40%), echogenic changes (fibrinous deposits on the serosal surface of the reticulum) with or without fluid inclusions in six cows (60%) and free fluid in the reticular area in one cow (10%). Abomasal dilatation was diagnosed in one of five cows (20%) and fibrinous changes and/or free fluid in the abomasal region in three of five cows (60%). Overall, ten (83%) of 12 cows had ultrasonographic evidence of localised or generalised peritonitis (Fig. 4).

**Table 3:** Ultrasonographic findings in 14 cows with type-5 abomasal ulcer

Location	Findings	Number of cows	Percent
Reticulum (n = 10)	Elevated from ventral abdominal wall	2	20
	Contour abnormal	4	40
	Amplitudes of contraction decreased	3	30
	Reticular atony	4	40
	Echogenic changes with or without fluid inclusions	6	60
	Free fluid in reticular region	1	10
Abomasum (n = 5)	Dilated	1	20
	Fibrin deposits on serosa	2	40
	Free fluid in abomasal region	1	20
Abdomen (n = 12)	Generalised echogenic lesions	1	8
	Generalised free fluid	6	50

### Concurrent diseases

Three cows (21%) had one concurrent disease and another cow had three concurrent diseases. Endometritis was diagnosed twice, and ketosis, fasciolosis, dicrocoeliosis and periarthritis once each.

### Diagnosis, treatment and euthanasia

A definitive diagnosis of U5 could not be made in any cow based on the clinical, laboratory and ultrasonographic findings. However, fibrin deposits on the serosa of the abomasum accompanied by ascites in two cows made a diagnosis of U5 or U4 likely. In seven other cows, a diagnosis of peritonitis presumably attributable to U4 or U5 was made based on the aspiration of an exudate and fluid accumulation in the abomasal region or abdomen. The differential diagnosis included peritonitis attributable to other causes including traumatic reticuloperitonitis or ruptured bowel. Five cows (36%) were euthanased immediately after examination because the results of clinical, laboratory or ultrasonographic examination led to a poor prognosis. One cow (7%) cow was euthanased after exploratory laparotomy because of severe untreatable changes, and eight cows (57%) were euthanased after unsuccessful treatment.

### Postmortem diagnosis

All cows underwent postmortem examination, which allowed the diagnosis of U5. All cows had a perforated abomasal ulcer and omental bursitis.

## Discussion

The clinical signs in cows with U5 are attributable primarily to omental bursitis, which has been described in textbooks [17, 19] and in studies [14–16, 18]. The lead signs vary widely and are associated with subacute to chronic peritonitis. Unfortunately, they are nonspecific; obtunded demeanour, indigestion, abdominal guarding and rumen atony were the most common clinical signs recorded in the present study. Abdominal distension observed in several cows with U5 [14, 16, 18] was not seen in our study; however, positive BSA and PSA on the left side in three cows and on the right side in nine cows, in the absence of displaced abomasum and diarrhoea, suggested an increased amount of abdominal fluid and gas. Of interest, only 46% of the cows had a only mild fever in spite of massive inflammation; in 39%, the rectal temperature was in the normal range and in 15% it was lower than normal. Similarly, only 43% of cows with traumatic reticuloperitonitis (TRP) [23], 49% of cows with U4 [10] and 58% of cows with U3 [12] had a mild fever, but 14% of cows with TRP [23], 20% of cows with U3 [12] and 30% of cows with U4 [10] had a high rectal temperature ranging from 39.6 to 41.3 °C. Possible reasons for normothermia or hypothermia seen in several cows with U5 include chronicity of the disease and, in cases with acute or subacute lesions, centralisation of the circulation as seen under shock conditions. Rumen atony was seen in 64% of cows with U5 compared with 49% of cows with U3 and 73% of cows with U4. In contrast, only 6% of cows with TRP had rumen atony. Complete rumen atony should be interpreted as a serious clinical finding. At least one of three tests for cranial abdominal pain was positive in 38% of cows with U5, which was in agreement with the findings in cows with U3 (45%) but considerably lower than those in cows with TRP or U4 (58%). Changes in faecal output and consistency are typical albeit nonspecific signs of omental bursitis; faecal output was reduced in 64% of cows and diarrhoea was seen in 50% of cows. Reduced or no faecal output was diagnosed in 77 and 79% of cows with U3 and U4, respectively, but in only 35% of cows with TRP, suggesting that abomasal ulcers have a more severe impact on intestinal motility than TRP. Dark brown or black manure in 21% of cows with U5 suggested haemorrhage of the ulcer, which occurred in a similar percentage of cows with U3 (10%) and U4 (16%). In contrast, melena was seen in 80% of cows with U2 [11] but in none of the cows with TRP. The most frequent sign of pain observed in cows with U5 was abdominal guarding, which was seen in all cows compared with 61 and 81% of cows with U3 and U4, respectively. Other pain manifestations were arched back in 36% of cows (TRP 14, U3 13, U4 28%), bruxism in 21% (16, 18, 25%, respectively) and spontaneous grunting in 14% (2, 0, 18%, respectively). In our experience, bruxism and spontaneous grunting only occur with severe pain and are therefore considered alarming findings. Seven of 14 cows with U5 had abnormal transrectal findings including an enlarged rumen, reduced intra-abdominal pressure and crepitus. Findings described in other reports, including a thickened greater omentum along the edge where the parietal lamina is reflected as the visceral lamina [17], an amorphous spongy mass [19] and a fluid-filled sac [16] were not found in our study.

The main reason for hypokalemia was most likely anorexia because forage is the main source of potassium [24]. Similar to cows with U3 (75%) and U4 (72%), hypokalemia occurred in 71% of cows with U5. Other causes of hypokalemia were discussed in detail [24]. Haemoconcentration was diagnosed in 57% of cows with U5, which was largely comparable to the rates in cows with U3 (35%) and U4 (69%) but considerably greater than in cows with TRP (12%) [10, 12, 23]. A high haematocrit reflects shock-associated haemoconcentration, but interestingly this was not accompanied by increased plasma protein concentration. With dehydration, an increase in haematocrit is accompanied by an increase in plasma protein concentration but in the present study, the plasma protein concentration was normal in 11 of 14 cows and lower than normal in the remaining three cows. Of 87 cows with a U4, 29% had a decrease in plasma protein concentration, whereas only 12% of cows with TRP had an increase in haematocrit and only 1% had a decrease in plasma protein concentration. A high haematocrit combined with a normal or lower-than-normal plasma protein concentration suggests active secretion of protein-rich fluid into the peritoneal cavity [25]. The importance of this laboratory abnormality as a diagnostic criterion for peritonitis is well established [26] reflecting the massive loss of fluid and protein into the omental bursa in cows with U5 or into the peritoneal cavity in cows with U4 as a result of severe inflammation. Protein-losing enteropathy should be included in the list of differential diagnoses in cattle with haemoconcentration associated with hypoproteinemia. Azotaemia occurred in 43% of cows with U5 and most likely reflected prerenal azotaemia. Similar to haemoconcentration, it represents an estimate of the severity of shock. The prevalence of azotaemia in cows with U3 (35%) and TRP (14%) was lower, whereas it was higher in cows with U4 (56%).

A definitive diagnosis of U5 could not be made in any cow. We believe that the diagnosis of U5 can be improved considerably through more elaborate ultrasonographic examination and routine abdominocentesis in all cows with ascites and/or ultrasonographic findings suggesting inflammatory changes such as fibrin deposits on serosal surfaces. The cows of the present study were examined over a period of 23 years, during which time ultrasonography has undergone tremendous improvements in terms of equipment and technique. It is now possible to differentiate inflammatory changes that are within and outside of the omental bursa via ultrasonography. Moreover, changes associated with omental bursitis involve primarily the left side of the abdomen.

## Conclusions

A reliable diagnosis of type-5 abomasal ulcer was not possible in the cows described in this paper. However, we believe that the diagnosis of U5 can be improved through thorough ultrasonographic examination, routine abdominocentesis in all cows with inflammatory abdominal changes and careful consideration of the pathological lesions typical of omental bursitis.

## Data Availability

The datasets used and analysed for this study are available from the corresponding author on reasonable request.
